# Prognostic relevance of programmed cell death 1 ligand 2 (PDCD1LG2/PD-L2) in patients with advanced stage colon carcinoma treated with chemotherapy

**DOI:** 10.1038/s41598-020-79419-3

**Published:** 2020-12-18

**Authors:** Kevin Chih-Yang Huang, Shu-Fen Chiang, Tsung-Wei Chen, William Tzu-Liang Chen, Pei-Chen Yang, Tao-Wei Ke, K. S. Clifford Chao

**Affiliations:** 1 Department of Biomedical Imaging and Radiological Science, China Medical University, Taichung, 40402 Taiwan; 2 Translation Research Core, China Medical University Hospital, China Medical University, Taichung, 40402 Taiwan; 3 Cancer Center, China Medical University Hospital, China Medical University, Taichung, 40402 Taiwan; 4 Lab of Precision Medicine, Feng-Yuan Hospital, Taichung, 42055 Taiwan; 5 Department of Pathology, Asia University Hospital, Taichung, 41354 Taiwan; 6 Graduate Institute of Biomedical Science, China Medical University, Taichung, 40402 Taiwan; 7 Department of Colorectal Surgery, China Medical University HsinChu Hospital, China Medical University, HsinChu, 302 Taiwan; 8 Department of Colorectal Surgery, China Medical University Hospital, China Medical University, Taichung, 40402 Taiwan; 9 Shool of Medicine, China Medical University, Taichung, 40402 Taiwan

**Keywords:** Cancer microenvironment, Colon cancer, Tumour biomarkers, Tumour immunology, Cancer, Biomarkers, Gastroenterology, Oncology

## Abstract

Colorectal cancer (CRC) is the leading cause of cancer-related mortality worldwide. Although the role of tumor programmed cell death 1 ligand 1 (PD-L1) in suppressing antitumor immunity has been validated in various malignances, the impact of PD-L2 (PD-L2/PDCD1LG2) within tumors remains elusive. Here, we examined tumor PD-L2 expression by immunohistochemical analysis and assessed its association with clinicopathological characteristics and the infiltration of intratumoral T lymphocytes in colon carcinoma patients (n = 1264). We found that tumor PD-L2 status was correlated with perineural invasion (PNI) and associated with survival outcome in colon carcinoma patients. The level of tumor PD-L2 was positively associated with tumor PD-L1 expression but inversely associated with the density of CD8^+^ tumor-infiltrating lymphocytes (TILs). Patients with elevated tumor PD-L2 levels had a favorable 5-year overall survival (OS) compared to patients with low PD-L2 levels (57% vs 40%, *p* < 0.001), especially in advanced stage colon carcinoma patients. Low tumor PD-L2 expression was associated with an increased 5-year OS risk among advanced stage colon carcinoma patients by univariate analysis [hazard ratio (HR) = 1.69, 95% CI 1.324–2.161, *p* < 0.001] and multivariate analysis [HR = 1.594, 95% CI 1.206–2.106, *p* = 0.001]. Moreover, tumor PD-L2 expression was inversely associated with the lymphocytic reaction in advanced stage colon carcinoma, suggesting that PD-L2 may be upregulated by a compensatory mechanism to inhibit T cell-mediated anticancer immunity. Taken together, these results show that tumor PD-L2 expression may be an independent prognostic factor for survival outcome in patients with advanced stage colon carcinoma.

## Introduction

Colorectal cancer (CRC) is the leading cause of cancer-related mortality worldwide, representing approximately 10% of cancer cases^1^. Despite advances in treatment for CRC patients, a remarkable proportion of CRC patients still develop tumor relapse, such as local recurrence (LR) and distant metastasis (DM), within 5 years after curative surgery^2,3^. Moreover, the survival of advanced-stage CRC is poor even when patients receive adjuvant chemotherapy. Thus, novel therapeutic strategies, such as immunotherapy strategies, need to be developed to improve the survival outcome of CRC patients.

The development and progression of CRC are triggered in a stepwise-manner by many genetic mutations in cancer cells as well as host-tumor interactions within the tumor microenvironment (TME)^4,5^. Anti-cancer immunity is indispensable for the surveillance and destruction of tumors; however, accumulating evidence indicates that immune checkpoint mechanisms downregulate anti-cancer immunity and lead to immune evasion within the TME^6^. Recently, antibodies targeting the immune checkpoint protein programmed cell death receptor 1 (PD1) and its ligand programmed cell death 1 ligand 1 (PD-L1/CD274) have clinically improved the survival outcomes in several malignances^7–9^. The therapeutic responses to immune checkpoint blockade are influenced by the molecular characteristics, immune cell profiles and tumor PD-L1 expression within the TME^4,10–13^. Although the infiltration of T lymphocytes is correlated with improved outcomes in CRC, the degree of T lymphocyte infiltration is determined by tumor antigens and tumor molecular characteristics such as microsatellite instability (MSI)^13–15^. Infiltrating T lymphocytes, such as cytotoxic CD8^+^ T cells, may induce adaptive immunity resistance by upregulating PD-L1 via effects on IFNγ to inhibit anti-tumor immunity^11,16^. However, the function of PD-L2 (PDCD1 ligand 2, PDCD1LG2), the other ligand for PD1, within the TME remains mostly unknown. A few reports have suggested that PD-L2 may be implicated in the induction of immune tolerance under physiological and pathological conditions^17,18^ and promote CD8^+^ T cell-mediated anti-tumor immunity^19^.

In this study, we examined the relationship between tumor PD-L2 expression and clinicopathological characteristics, such as DNA mismatch repair status as well as immune cell profiles, and elucidated the prognostic value of tumor PD-L2 expression in colon carcinomas. We found that patients with elevated tumor PD-L2 levels had favorable 5-year survival outcomes in advanced stage colon carcinoma compared with patients with low tumor PD-L2 expression, which is consistent with the results from The Cancer Genome Atlas (TCGA)^20^, especially in patients who received chemotherapy. Encouragingly, we found that tumor PD-L2 status was inversely correlated with the density of intratumoral CD8^+^ TILs, suggesting that the level of PD-L2 was not mainly induced by IFN-γ from CD8^+^ TILs but by other cytokines within the tumor microenvironment. These findings indicate that PD-L2 may be an independent prognostic factor for advanced stage colon carcinoma patients.

## Materials and methods

### Patient characteristics

Colon carcinoma patients (n = 1264; stage I: 175, stage II: 456, stage III: 406, and stage IV: 227) who underwent surgery at the Department of Colorectal Surgery of China Medical University Hospital between 2006 and 2014 were enrolled in this study (Table 1). The average age of the patients was 63.1 years, with a range of 23 to 93 years. Postoperative pathohistological analysis was performed before enrollment in this study, and patients were classified according to the tumor-node-metastasis staging system (AJCC 7th edition staging). A postoperative chemotherapeutic regimen was recommended for high-risk stage II patients and lymph node metastasis stage III patients identified in surgical specimens according to the status of the patients (Table S1).

**Table 1:** Tumor characteristics in colon carcinoma patient (n = 1264).

Clinicopathological parameters	Total no.	Tumor PD-L2		*p* value
		High	Low	
	1264	390	874	
**Sex**				0.513
Female	579 (45.8%)	184 (47.2%)	395 (45.2%)	
Male	685 (54.2%)	206 (52.8%)	479 (54.8%)	
**Age**				0.024*
< 65	598 (47.3%)	166 (42.6%)	432 (49.4%)	
≥ 65	666 (52.7%)	224 (57.4%)	442 (50.6%)	
**Tumor location**				0.052
Proximal colon	575 (45.6%)	161 (41.3%)	414 (47.3%)	
Distal colon	677 (53.5%)	224 (57.6%)	453 (51.8%)	
Unspecified	12 (0.9%)	5 (1.3%)	7 (0.9%)	
**Pathological TNM stage**				< 0.001*
Stage I	175 (13.8%)	44 (11.3%)	131 (15.0%)	
Stage II	456 (36.1%)	150 (38.5%)	306 (35.0%)	
Stage III	406 (32.1%)	150 (38.5%)	256 (29.3%)	
Stage IV	227 (18.0%)	46 (11.8%)	181 (20.7%)	
**Lymphovascular invasion (LVI)**				0.213
Absent	596 (47.2%)	194 (49.7%)	402 (46.0%)	
Present	662 (52.4%)	194 (49.7%)	468 (53.5%)	
Unknown	6 (0.5%)	2 (0.5%)	4 (0.5%)	
**Perineural invasion (PNI)**				< 0.001*
Absent	757 (59.9%)	201 (50.1%)	556 (63.6%)	
Present	499 (39.5%)	186 (47.7%)	313 (35.8%)	
Unknown	8 (0.6%)	3 (0.8%)	5 (0.6%)	
**Tumor differentiation**				0.451
Well to moderate	1233 (97.5%)	383 (98.2%)	850 (97.3%)	
Poor	10 (0.8%)	2 (0.5%)	8 (0.9%)	
Unknown	21 (1.7%)	5 (1.3%)	16 (1.8%)	
**MMR status**				< 0.001*
MMR-proficient	1167 (92.3%)	385 (98.7%)	782 (89.5%)	
MMR-deficient	96 (7.6%)	5 (1.3%)	91 (10.4%)	
NA	1 (0.1%)	0 (0%)	1 (0.1%)	

NA: not available. The Fisher's exact test did not include the “NA” and "unknown" group.

*indicated *p*<0.05.

### Tissue microarray (TMA) construction and immunohistochemistry (IHC)

The resected tumor tissue and corresponding normal mucosa specimens from primary tumors of colon carcinoma patients before chemotherapy were collected to establish a TMA (Table 1). The protocol for the establishment of TMA has been previously described^13,21^ and was approved by the Institutional Review Board (IRB) of China Medical University Hospital [protocol number: CMUH105-REC2-073]. Briefly, tumor areas were evaluated on hematoxylin/eosin (HE)-stained tissue slides by a pathologist (Dr. Tsung-Wei Chen), and the corresponding area on a formalin-fixed, paraffin-embedded (FFPE) tissue block (donor block) was identified for TMA construction. Tissue cylinders (2 mm in diameter) were punched from the central tumor tissue areas from each donor block, and these individual tissue cylinders were placed into one recipient paraffin block. Each TMA spot contained at least 50% of the tumor area.

Immunohistochemical staining was performed on 3-μm-thick TMA sections according to the manufacturer’s manual (VECTASTAIN Elite ABC Kit, Vector Laboratories, CA, USA), and the TMAs were incubated with DAB (Vector Laboratories) and then counterstained with hematoxylin^22,23^. Positive staining for CD8 (ab4055, Abcam, Cambridge, UK), CD45RO (ab23, Abcam) and PD1 (ab52587, Abcam) was defined as staining in the cytoplasm or membranous intratumoral TILs by microscopy (Olympus BX53, Tokyo, Japan). Evaluation of TILs in TMA tissues was performed at 400 × magnification by a pathologist. The area with the highest density of TILs within the TME was counted at 400 × magnification [No. of positively stained TILs/high-power field (HPF)]. The average count of TILs in five HPFs was scored: a count of 0 positively stained TILs in one HPF was scored as 0, 1–3 positively stained TILs/HPF was scored as 1, 4–10 positively stained TILs/HPF was scored as 2, and > 10 positively stained TILs/HPF was scored as 3^13^.

Tumor PD-L2 (ab200377, Abcam) expression was evaluated based the extent and intensity of immunopositivity of tumor cells as assessed by a semiquantitative scale (0–3+) as follows: 0 for absent; 1 for weak; 2 for moderate; and 3 for strong membrane staining. The percentage of positive cells was scored as follows: 0–10% positive tumor cell proportion was scored as 0; 10–25% positive tumor cell proportion was scored as 1; 26–50% positive tumor cell proportion was scored as 2; and 51%-100% positive tumor cell proportion was scored as 3. The product of the two scores served as the tumor PD-L2 immunostaining score. The cutoff for the immunostaining score was 4^13,15^. The percentage of cancer cells with membranous PD-L1 (ab205921) staining was scored as follows: 0 points were assigned when < 5% positive tumor cells were detected, and 1 point was assigned when > 5% of cells showed positive membranous staining^24^. To evaluate the status of mismatch repair (MMR), MMR proficiency was defined the expression of 4 proteins: MLH1 (ab92312, Abcam), MSH2 (ab92372, Abcam), MSH6 (ab92471, Abcam) and PMS2 (ab110638, Abcam), while MMR-deficient tumors were defined as those lacking at least one of these four markers by IHC.

### The Cancer Genome Atlas (TCGA) database

A total of 259 patients with stage III (n = 174) and IV (n = 85) colorectal cancer were included, and their *PD-L2* mRNA expression data were retrieved from the open-access Human Pathology Atlas resource, which is part of the Human Protein Atlas (HPA, www.proteinatlas.org/pathology)^20,25^; HPA contains RNA sequencing (RNA-seq) data together with clinical information from TCGA. The *PD-L2* mRNA level had the best log-rank *p* value based on the Kaplan–Meier analysis. The average RNA expression level was utilized as the cutoff (high expression cutoff = 0.21) according to the algorithm on the HPA website^20^.

### Statistical analysis

SPSS (IBM SPSS Statistics 22, WA, USA) and GraphPad Prism 7 (GraphPad Software, CA, USA) were utilized to perform statistical analysis in the study. All tests were analyzed with two-sided *p*-values with the significance level set at 0.05, including Student’s t-test and Pearson’s chi-square test. Univariate and multivariate models were analyzed by Cox regression analysis with hazard ratios (HRs) and 95% confidence intervals (CIs)^26^. The influential factors were included in the Cox models, including sex (male vs female), age (≥ 65 years vs < 65 years), pT stage (pT3-4 vs pT1-2), pN stage (positive vs negative), pTNM stage (stage IV vs stage III), lymphovascular invasion (presence vs absence), perineural invasion (presence vs absence), tumor location (proximal colon vs distal colon), CD8^+^ TIL density (high vs low), CD45^+^ TIL density (high vs low), CD45RO^+^ TIL density (high vs low), PD1^+^ TIL density (high vs low), tumor PD-L1 level (high vs low) and tumor PD-L2 level (high vs low). The 5-year overall survival (OS) was estimated by the Kaplan–Meier method and defined as the duration between the day of diagnosis and the day of death. Log-rank tests were performed for univariate comparisons.

### Ethical approval

This study was reviewed and approved by the Internal Review Board (IRB) of China Medical University Hospital [Protocol number: CMUH105-REC2-073]. The method was carried out in accordance with the committee’s approved guidelines.

### Informed consent

Informed consents were obtained from all participants in the study.

## Results

### Immunohistochemical expression of tumor PD-L2, clinicopathological characteristics and the status of MMR in stage I-IV colon carcinoma

**Figure 1:**
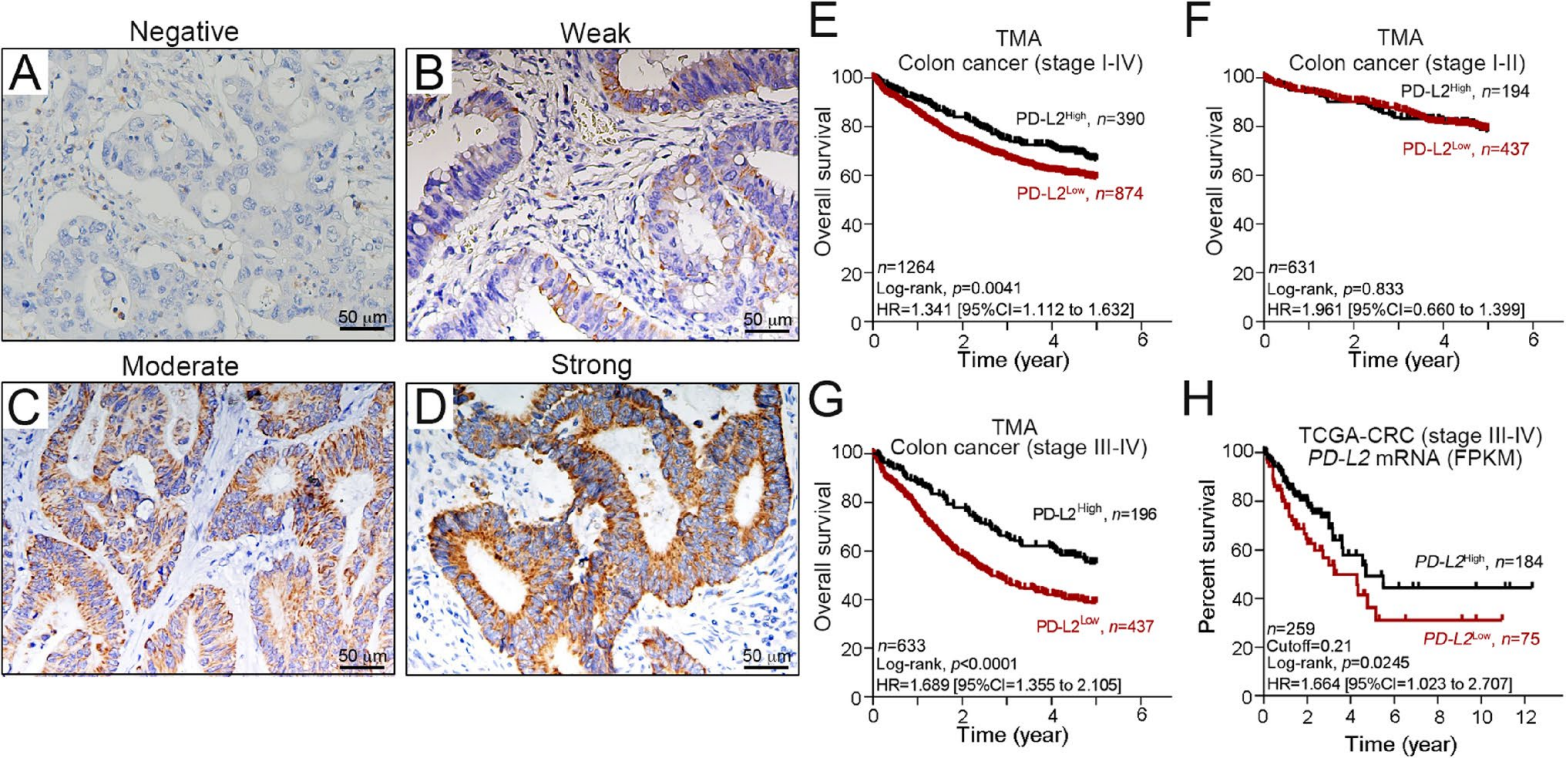
Expression patterns of tumor PD-L2 within the tumor microenvironment are associated with survival outcome in stage III-IV colon carcinoma. (**A**) Negative tumor PD-L2 immunohistochemical staining within the tumor microenvironment. (**B**) Weak cytoplasmic tumor PD-L2 expression. (**C**) Moderate cytoplasmic tumor PD-L2 expression. (**D**) Strong cytoplasmic tumor PD-L2 expression. Scale bar = 50 μm. (**E**) High tumor PD-L2 was positively associated with 5-year overall survival in our TMA cohort (stage I–IV, *n* = 1264, *p* = 0.0041). (**F**) Tumor PD-L2 was not associated with 5-year overall survival in early-stage colon carcinoma (stage I–II, *n* = 631, *p* = 0.833). (**G**) High tumor PD-L2 was positively associated with 5-year overall survival in our TMA cohort (stage III–IV, *n* = 633, *p* < 0.001). (**H**) High *PD-L2* mRNA was positively associated with 5-year survival outcome in the stage III–IV CRC cohort from TCGA (stage III–IV, *n* = 259, *p* = 0.0245).

To evaluate the clinical impact of tumor PD-L2 in stage I-IV colon carcinoma, we used immunohistochemical analysis to detect the protein expression of tumor PD-L2 (n = 1264, Table 1). We scored tumor PD-L2 expression in the cytoplasm and membrane based on the intensity of staining and the proportion of cells with positive staining^13^ (Fig. 1). Representative images of immunohistochemical PD-L2 expression in tumor cells are shown according to their staining intensity (Fig. 1A–D). Patient clinicopathological characteristics and tumor PD-L2 expression are summarized in Table 1.

We found that 30.9% (390/1264) of colon carcinoma patients overall and 7.6% (96/1264) of MMR-deficient patients had high expression PD-L2 in the tumor bed. The expression of tumor PD-L2 within the TME was significantly associated with the absence of perineural invasion (PNI, *p* < 0.001) and MMR proficiency (*p* < 0.001, Table 1).

### Elevated tumor PD-L2 expression is significantly associated with favorable 5-year OS in advanced stage colon carcinoma

We used Kaplan–Meier (KM) analysis to examine the prognostic role of tumor PD-L2 expression in stage I-IV colon carcinoma. The KM survival curve showed that high tumor PD-L2 expression versus low tumor PD-L2 expression was significantly associated with favorable 5-year OS in stage I-IV patients (67% vs 60%, *p* = 0.0041, Fig. 1E). To further determine the role of tumor PD-L2 in colon carcinoma, we stratified colon carcinoma patients into early-stage (stage I-II) and advanced stage (stage III-IV) subgroups based on TNM stage. We found that the status of tumor PD-L2 was not significantly associated with survival outcome in early-stage colon carcinoma patients (79.4% vs 79.6%, *p* = 0.833, Fig. 1F), patients without PNI (*p* = 0.014) or MMR-proficient patients (*p* = 0.001, Table 2). However, KM curves and log-rank tests revealed that high expression of tumor PD-L2 was associated with a remarkably longer 5-year OS than low expression of tumor PD-L2 in advanced stage patients (57% vs 40%, *p* < 0.001, Fig. 1G). We then analyzed *PD-L2* mRNA data from The Cancer Genome Atlas (TCGA) (stage III-IV, n = 259, Fig. 1H)^20^. Data on *PD-L2* mRNA expression in stage III-IV colorectal cancer samples were retrieved from the Human Protein Atlas (HPA, www.proteinatlas.org/pathology) ^20,25^, which employs RNA sequencing (RNA-seq) data together with clinical information from TCGA. The cutoff for the *PD-L2* mRNA level for KM analysis was based on the algorithm on the HPA website ^20^. CRC patients with high *PD-L2* mRNA had favorable survival outcomes.

**Table 2:** Tumor characteristics in late-stage colon carcinoma patient (n = 633).

Clinicopathological parameters	Total no	Tumor PD-L2		*p* value
		High	Low	
	633	196	437	
**Sex**				0.704
Female	290	92 (46.9%)	198 (45.3%)	
Male	343	104 (53.1%)	239 (54.7%)	
**Age**				0.067
< 65	325	90 (45.9%)	235 (53.8%)	
≥ 65	308	106 (54.1%)	202 (46.2%)	
**Tumor location**				0.274
Proximal colon	283	94 (48%)	189 (43.2%)	
Distal colon	343	100 (51%)	243 (55.6%)	
Unspecified	7	2 (1.0%)	5 (1.1%)	
**pT stage**				0.828
pT1-2	40	13 (6.6%)	27 (6.2%)	
pT3-4	593	183 (93.4%)	410 (93.8%)	
**pN stage**				0.563
Negative	27	7 (3.6%)	20 (4.6%)	
Positive	606	189 (96.7%)	417 (95.4%)	
**M stage**				< 0.001*
M0	406	150 (76.5%)	256 (58.6%)	
M1	227	46 (23.5%)	181 (41.4%)	
**Pathological TNM stage**				< 0.001*
Stage III	406	150 (76.5%)	256 (58.6%)	
Stage IV	227	46 (23.5%)	181 (41.4%)	
**Tumor differentiation**				0.799
Well to moderate	615	190 (96.9%)	425 (97.3%)	
Poor	4	1 (0.5%)	3 (0.7%)	
Unknown	14	5 (2.6%)	9 (2.1%)	
**Lymphovascular invasion (LVI**)				0.18
Absent	146	52 (26.5%)	94 (21.5%)	
Present	484	144 (73.5%)	340 (77.8%)	
Unknown	3	0 (0%)	3 (0.7%)	
**Perineural invasion (PNI)**				0.014*
Absent	267	69 (35.2%)	198 (45.3%)	
Present	363	127 (64.8%)	236 (54.0%)	
Unknown	3	0 (0%)	3 (0.7%)	
**Post-operative chemotherapy**				0.942
No	228	71 (36.2%)	157 (35.9%)	
Yes	405	125 (63.8%)	280 (64.1%)	
**MMR status**				0.001*
MMR-proficient	601	196 (100%)	405 (92.7%)	
MMR-deficient	31	0 (0%)	31 (7.1%)	
NA	1	0 (0%)	1 (0.2%)	

NA: not available. The Fisher's exact test did not include the “NA” and "unknown" group.

*indicated *p*<0.05.

**Figure 2:**
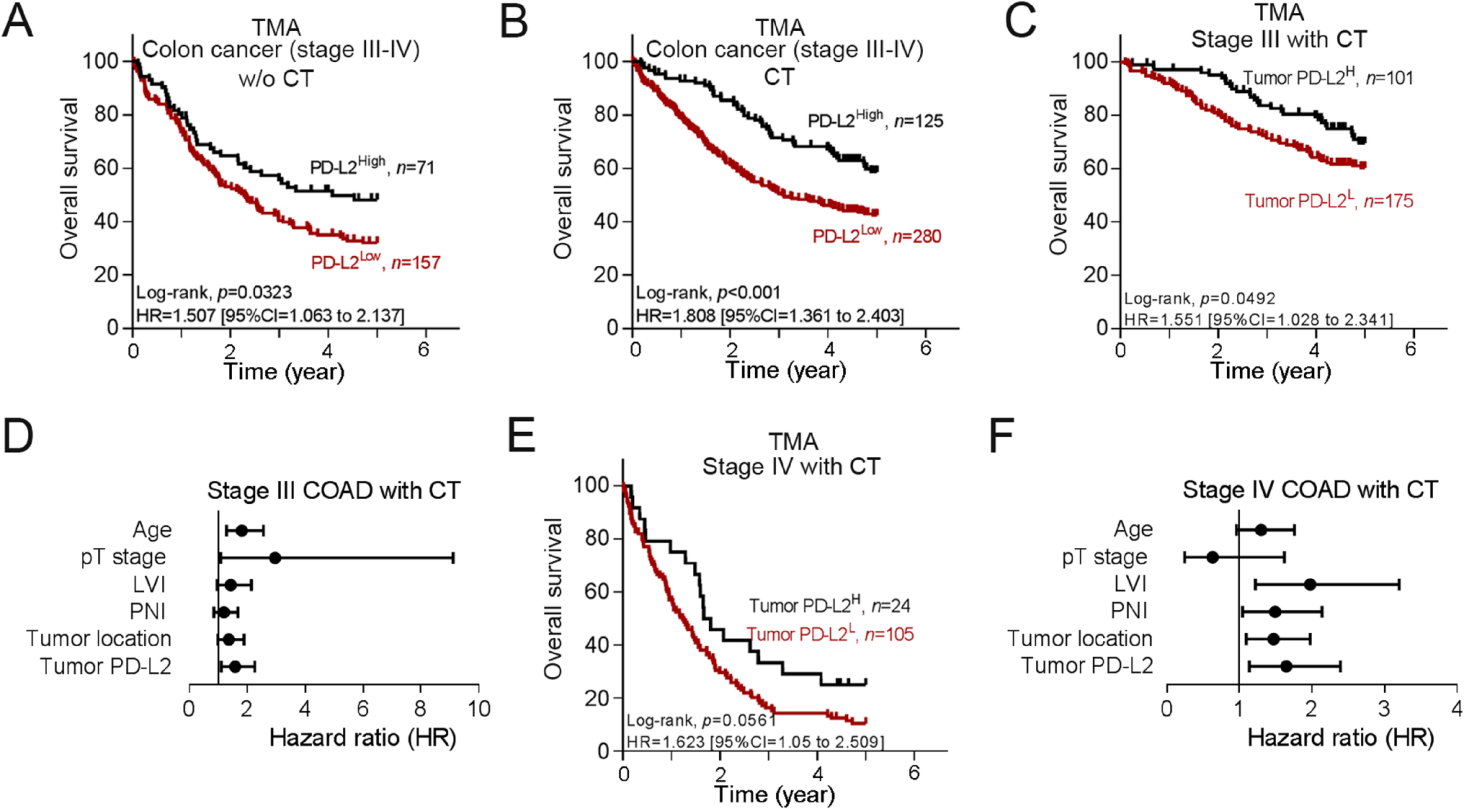
High tumor PD-L2 is remarkably associated with favorable 5-year OS in patients who receive postoperative chemotherapy. (**A**) High tumor PD-L2 was associated with better 5-year OS in advanced stage colon carcinoma patients (*n* = 228, *p* = 0.0323). CT: chemotherapy. (**B**) High tumor PD-L2 levels were remarkably associated with favorable 5-year OS in advanced stage colon carcinoma patients who received postoperative chemotherapy (*n* = 405, *p* < 0.001). (**C**) Patients with high tumor PD-L2 level was significantly associated with better 5-year OS in stage III colon carcinoma patients who received adjuvant chemotherapy. CT: chemotherapy (*n* = 276, *p* = 0.0492). (**D**) The multivariate COX regression model showed tumor PD-L2 is an independent factor in 5-year OS of stage III COAD patients who received post-operative chemotherapy. The hazard ratios (HR) and 95% confidence interval (CI) of different factors in the COX regression model. (**E**) Patients with high tumor PD-L2 level were tendency to associate with better 5-year OS in stage IV colon carcinoma patients who received palliative chemotherapy. CT: chemotherapy (*n* = 129, *p* = 0.0561). (**F**) The multivariate COX regression model suggested tumor PD-L2 is an independent factor in 5-year OS of stage IV COAD patients who received post-operative palliative chemotherapy. The HR and 95% CI of different factors in the COX regression model.

Moreover, the KM analysis of clinicopathologic characteristics in advanced stage colon carcinoma showed that older age (*p* = 0.02), larger pathological tumor size (pT stage, *p* < 0.001), lymphovascular invasion (LVI, *p* < 0.001), PNI (*p* < 0.001) and proximal colon location (*p* = 0.021) were associated with shorter 5-year OS in advanced stage colon carcinoma (Table 3). Classifying advanced stage colon carcinoma patients into subgroups based on stage and postoperative chemotherapy regimen, we found that stage III or IV patients with elevated tumor PD-L2 tended to have better 5-year OS than those with low tumor PD-L2 (Fig. S1A-1B and Table S2). Patients with advanced stage colon cancer who received postoperative chemotherapy with high tumor PD-L2 had more favorable 5-year OS than those with low tumor PD-L2 (Fig. 2A,B). Moreover, high tumor PD-L2 was clinically associated with improved 5-year OS in stage III (*p* = 0.0492, Fig. 2C) and stage IV (*p* = 0.0561, Fig. 2E) colon carcinoma patients who received postoperative chemotherapy. Multiverse analysis adjusted with clinicopathologic parameters suggested that tumor PD-L2 may be an independent prognostic factor for advanced stage colon carcinoma patients, especially for patients who receive postoperative chemotherapy (Stage III: HR 1.575, 95% CI 1.098–2.259, *p* = 0.045, Fig. 2D; Stage IV: HR 1.649, 95% CI 1.136–2.393, *p* = 0.008, Fig. 2F).

**Table 3:** Correlation between clinicopathologic parameters and 5-year OS (n = 633).

Parameters	No^a^	5-yr OS (%)	*p* value*
	633	45%	
**Sex**			0.388
Female	290	46%	
Male	343	45%	
**Age**			0.02*
< 65	325	49%	
≥ 65	308	42%	
**pT stage**			< 0.001*
pT1-2	40	78%	
pT3-4	593	43%	
**pN stage**			0.067
Negative	27	30%	
Positive	606	46%	
**M stage**			< 0.001*
M0	406	65%	
M1	227	13%	
**Pathological TNM stage**			< 0.001*
Stage III	406	65%	
Stage IV	227	13%	
**Lymphovascular invasion (LVI)**			< 0.001*
Absent	146	63%	
Present	484	40%	
**Perineural invasion (PNI)**			< 0.001*
Absent	267	58%	
Present	363	36%	
**Tumor location**			0.021*
Distal colon	343	49%	
Proximal colon	283	41%	
**Tumor differentiation**			0.189
Well to moderate	615	46%	
Poor	4	25%	
**Tumor PD-L1**			< 0.001*
High	234	56%	
Low	399	39%	
**Tumor PD-L2**			< 0.001*
High	196	57%	
Low	437	40%	

^a^Number of cases may differ due to missing data.

*indicated *p*<0.05.

### Tumor PD-L2 expression correlates inversely with intratumoral CD8^+^ TILs

**Figure 3:**
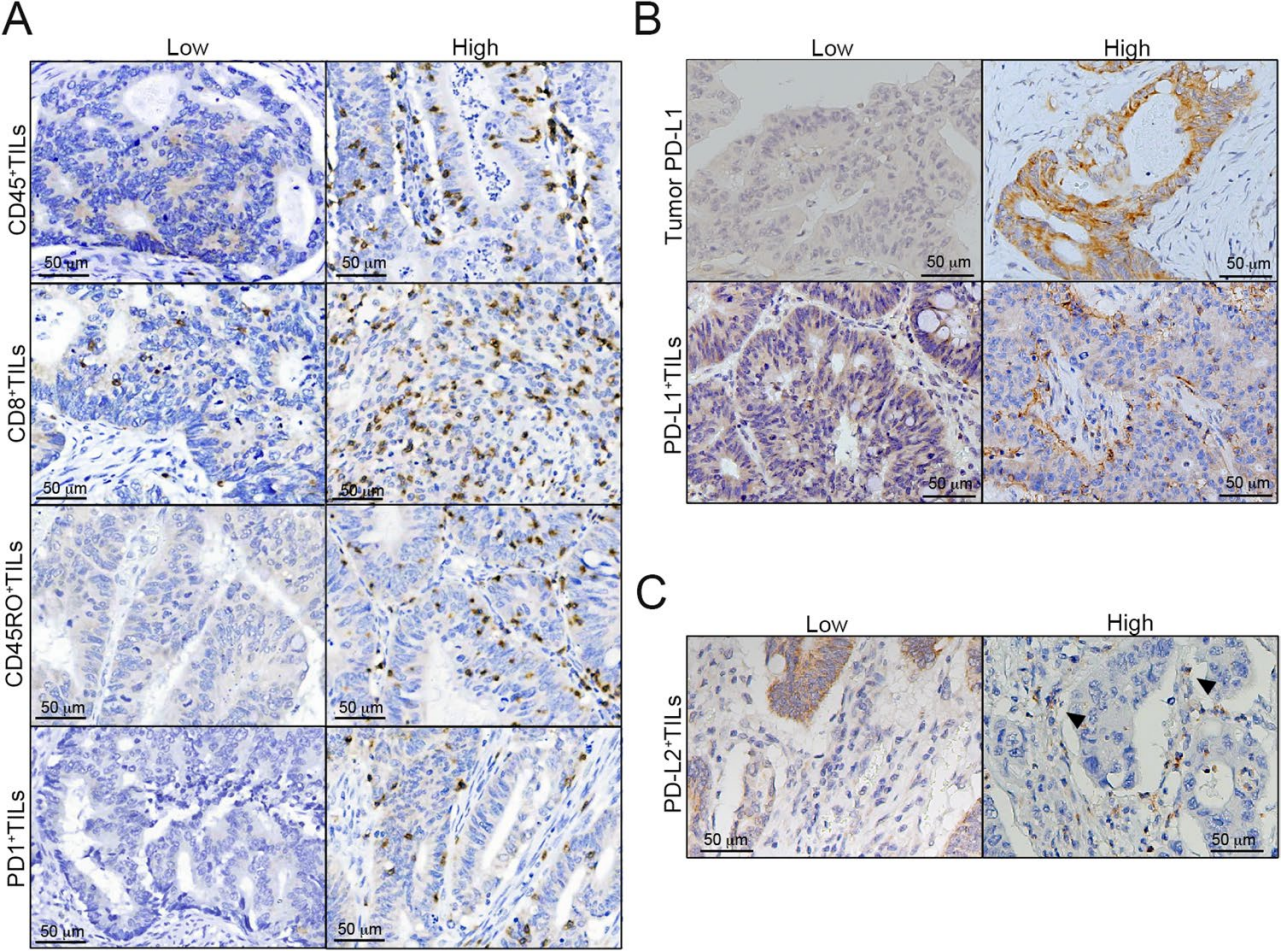
The representative images of immune signatures. (**A**) The intratumor infiltration of CD45^+^ cells (CD45 is a general lymphocyte marker), CD8^+^ cells (CD8 is a cytotoxic T lymphocyte marker), CD45RO^+^ cells (CD45RO is a memory T lymphocyte marker), PD1^+^ cells (PD1 is an immunosuppressive lymphocyte marker) within TME. (**B**) PD-L1 expression on tumor cells and intraepithelial TILs within TME. (**C**) PD-L2 was partially expressed in intraepithelial TILs within TME. Scale bar = 50 μm.

Our previous studies demonstrated a positive correlation between tumor PD-L1 and intratumoral CD8^+^ TILs in colorectal cancer^13,24,27^. Therefore, we evaluated the association between tumor PD-L2 and immune signatures such as infiltration of CD45^+^ cells (CD45 is a general lymphocyte marker), CD8^+^ cells (CD8 is a cytotoxic T lymphocyte marker), CD45RO^+^ cells (CD45RO is a memory T lymphocyte marker), PD1^+^ cells (PD1 is an immunosuppressive lymphocyte marker) and PD-L1 by immunohistochemical analysis of intraepithelial tumor-infiltrating lymphocytes within tumor beds (Fig. 3A). We found PD-L1 and PD-L2 partially expressed in lymphocytes (Fig. 3B,C). There was no statistically significant association of tumor PD-L2 expression with the infiltration of CD45^+^ TILs or CD45RO^+^ TILs (Table 4 and Table S3). Intriguingly, we found a direct negative correlation between tumor PD-L2 and the density of CD8^+^ TILs and PD1^+^ TILs (Tables 4 and Table S3). Patients (33/196; 16.8%) with high tumor PD-L2 had a high density of CD8^+^ TILs, and 105 of 437 (24.0%) patients with low tumor PD-L2 had a high number of CD8^+^ TILs, indicating that tumor PD-L2 expression was inversely correlated with intratumoral CD8^+^ TILs (*p* = 0.048, Pearson's chi-squared test). Moreover, tumor PD-L2 positively correlated with the status of tumor PD-L1 (*p* < 0.001, Pearson's chi-squared test, Table S3). Tumor PD-L1 expression positively correlated with the immune signature of CD8^+^ TILs (*p* < 0.001, Pearson's chi-squared test, Table S3).

**Table 4:** Tumor characteristics and immune status in late-stage colon carcinoma patient (n = 633).

Clinicopathological parameters	Total no	Tumor PD-L2		*p* value	Tumor PD-L1		*p* value
		High	Low		High	Low	
	633	196	437		234	399	
**CD8**^**+**^**TILs**				0.048*			< 0.001*
High	138	33 (16.8%)	105 (24.0%)		74 (31.6%)	64 (16.0%)	
Low	492	161 (82.2%)	331 (75.8%)		159 (68.0%)	333 (83.5%)	
Unknown	3	2 (1%)	1 (0.2%)		1 (0.4%)	2 (0.5%)	

NA: not available. The Fisher's exact test did not include the “NA” and "unknown" group.

*indicated *p*<0.05.

**Figure 4:**
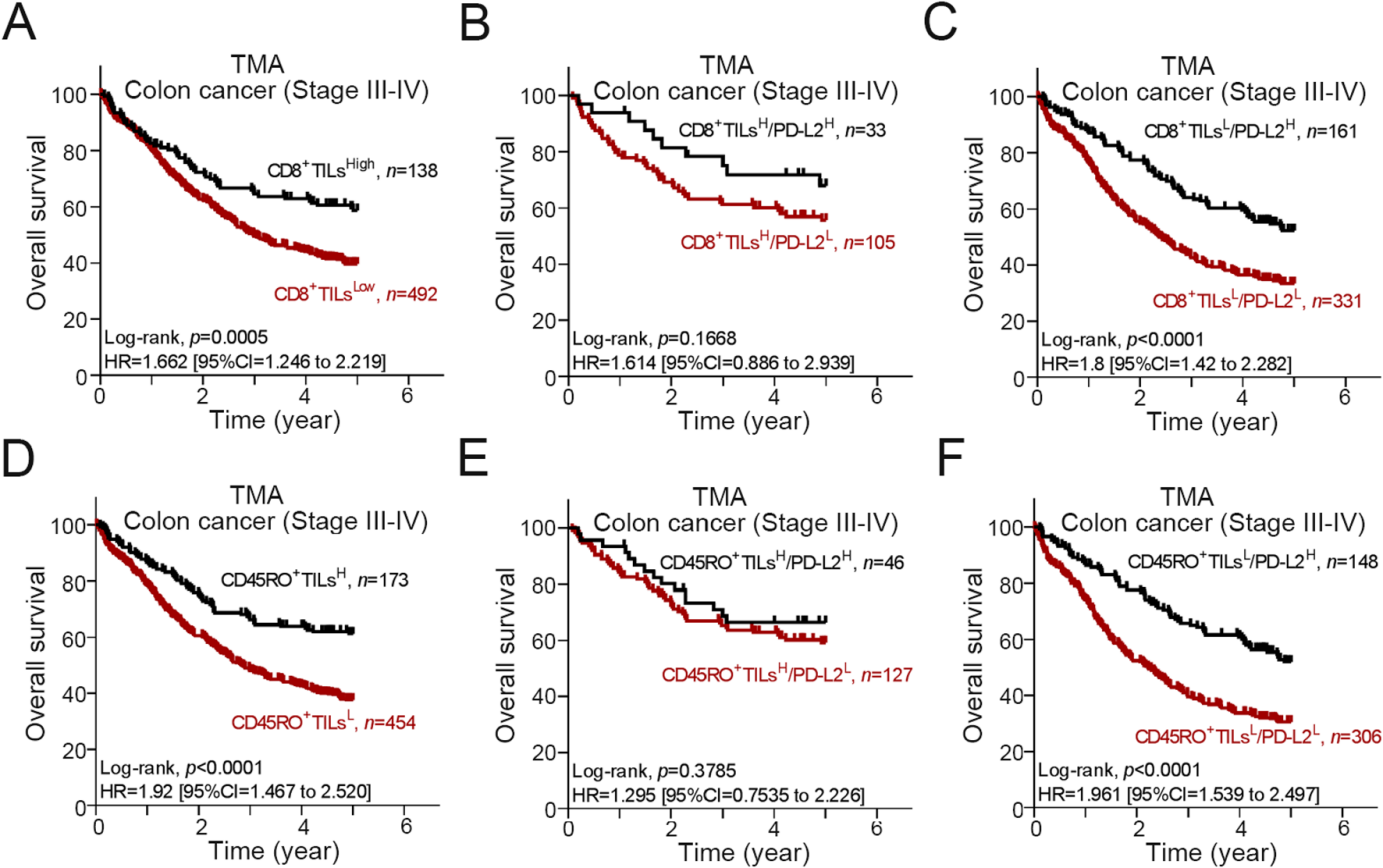
The association of tumor PD-L2 level and CD8^+^ TIL and CD45RO^+^ TIL density with 5-year OS in advanced stage colon carcinoma. (**A**) Advanced stage colon carcinoma patients with high infiltration of intratumoral CD8^+^ TIL within the TME had a better 5-year OS (n = 630, *p* = 0.0005). (**B**) The tumor PD-L2 level was not associated with 5-year OS in patients with a high density of CD8^+^ TILs within the TME (*n* = 138, *p* = 0.167). (**C**) The tumor PD-L2 level was associated with 5-year OS in patients with a low density of CD8^+^ TILs within the TME (*n* = 492, *p* < 0.001). (**D**) Advanced stage colon carcinoma patients with a high density of CD45RO^+^ TILs within the TME had a better 5-year OS (n = 627, *p* < 0.001). (**E**) The tumor PD-L2 level was not associated with 5-year OS in patients with a high density of CD45RO^+^ TILs within the TME (*n* = 173, *p* = 0.3785). (**F**) The tumor PD-L2 level was associated with 5-year OS in patients with a low density of CD45RO^+^ TILs within the TME (*n* = 454, *p* < 0.001).

To further clarify the role of TILs and tumor PD-L2 expression in CRC prognosis, we evaluated the association of patient survival outcomes with the density of cytotoxic CD8^+^ TILs, the density of memory CD45RO^+^ TILs and tumor PD-L2. Patients with high CD8^+^ TIL density within the TME had improved 5-year OS (log-rank, n = 630, *p* = 0.0005, Fig. 4A). We considered intratumoral CD8^+^ TILs and tumor PD-L2 expression in combination, and we found that tumor PD-L2 status was not associated with 5-year OS in the subgroup of patients with high CD8^+^ TILs (CD8^+^ TILs^H^/PD-L2^H^: 69.7% vs CD8^+^ TILs^H^/PD-L2^L^: 57.1%, *p* = 0.167, Fig. 4B). Intriguingly, the expression of tumor PD-L2 was significantly associated with 5-year OS in patients with low CD8^+^ TILs (CD8^+^ TILs^L^/PD-L2^H^: 54.0% vs CD8^+^ TILs^L^/PD-L2^L^: 35.0%, *p* < 0.001, Fig. 4C). Similarly, patients with high CD45RO^+^ TIL infiltration within the TME had improved 5-year OS (log-rank, n = 630, p < 0.0001, Fig. 4D). Tumor PD-L2 was not associated with 5-year OS in patients with high CD45RO^+^ TILs (Fig. 4E, p = 0.3785) but was significantly associated with 5-year OS in patients with low CD45RO^+^ TILs (Fig. 4F, p < 0.001). These results suggest that tumor PD-L2 expression can be a significant prognostic factor for advanced stage colon carcinoma patients, especially for patients with low densities of infiltrating lymphocytes.

### Prognostic impacts of tumor PD-L2 expression in advanced stage colon carcinoma

In univariate analysis of 5-year OS by the Cox regression model, the following clinicopathological characteristics were associated with patient survival outcome: age, pT stage, pN stage, pTNM stage, LVI, PNI and tumor location. Furthermore, the densities of infiltrating immune cells were also associated with the patient survival outcome: CD8^+^ TIL density, CD45^+^ TIL density, CD45RO^+^ TIL density, PD1^+^ TIL density, tumor PD-L1 and tumor PD-L2. Patients with a low density of immune infiltration had a higher risk for a low 5-year OS: low CD8^+^ TIL density (HR 1.66, 95% CI 1.246–2.219, *p* = 0.001), low CD45^+^ TIL density (HR 1.72, 95% CI 1.339–2.214, *p* < 0.001), low CD45RO^+^ TIL density (HR 1.92, 95% CI 1.467–2.520, *p* < 0.001), and low PD1^+^ TIL density (HR 2.21, 95% CI 1.510–3.232, *p* < 0.001). Moreover, patients with a low tumor PD-L2 also had an increased risk for a low 5-year OS (HR 1.69, 95% CI 1.324–2.161, *p* < 0.001) compared with patients with high tumor PD-L2 expression (Table 5). These results showed that immune factors such as CD8^+^ TILs, CD45^+^ TILs, CD45RO^+^ TILs, PD1^+^ TILs and tumor PD-L2 expression have clinical prognostic relevance for advanced stage colon carcinoma patients.

**Table 5:** Univariate and multivariate analysis of overall survival and known prognostic factors in late-stage colon carcinoma patient (n = 633).

Parameters	Univariate analysis					Multivariate analysis		
	No. at risk^a^	Deaths	HR	95% CI	*p* value	HR	95% CI	*p* value
**Sex**					0.39			
Female	290	156	1.00			–		
Male	343	190	1.10	0.888–1.357		–		
**Age**					0.02*			< 0.001*
< 65	325	166	1.00			1.00		
≥ 65	308	180	1.28	1.040–1.585		1.719	1.377–0.145	
**pT stage**					< 0.001*			0.194
T1–2	40	9	1.00			1.00		
T3–4	593	337	3.25	1.674–6.297		1.576	0.793–3.131	
**pN stage**					0.07			0.161
Negative	27	18	1.00			1		
Positive	606	327	0.65	0.410–1.035		0.687	0.407–1.161	
**M stage**					< 0.001*			
M0	406	148	1.00			–		
M1	227	198	4.71	3.782–5.861		–		
**Pathological TNM stage**					< 0.001*			< 0.001*
Stage III	406	148	1.00			1.00		
Stage IV	227	198	4.71	3.782–5.861		4.619	3.562–5.990	
**Lymphovascular invasion (LVI)**					< 0.001*			0.004*
Absent	146	54	1.00			1.00		
Present	484	291	2.00	1.499–2.681		1.604	1.168–2.203	
**Perineural invasion (PNI)**					< 0.001*			0.041*
Absent	267	111	1.00			1.00		
Present	363	234	1.87	1.493–2.347		1.299	1.011–1.670	
**Tumor location**					0.022*			0.001*
Distal colon	343	175	1.00			1.00		
Proximal colon	283	166	1.28	1.037–1.586		1.462	1.175–1.819	
**CD8**^**+**^**TILs**					0.001*			0.563
High	138	55	1.00			1.00		
Low	492	289	1.66	1.246–2.219		1.125	0.754–1.680	
CD45^+^TILs					< 0.001*			0.445
High	195	79	1.00			1.00		
Low	436	266	1.72	1.339–2.214		0.88	0.633–1.222	
CD45RO^+^TILs					< 0.001*			0.245
High	173	65	1.00			1.00		
Low	454	276	1.92	1.467–2.520		1.233	0.866–1.754	
**PD1**^**+**^**TILs**					< 0.001*			0.269
High	91	29	1.00			1.00		
Low	540	316	2.21	1.510–3.232		1.316	0.809–2.140	
**Tumor PD-L1**					< 0.001*			0.465
High	234	104	1.00			1.00		
Low	399	242	1.57	1.245–1.937		0.903	0.688–1.186	
**Tumor PD-L2**					< 0.001*			0.001*
High	196	85	1.00			1.00		
Low	437	231	1.69	1.324–2.161		1.594	1.206–2.106	

^a^Number of cases may differ due to missing data.

*indicated *p*<0.05.

Moreover, when these parameters that were identified as significant by the univariate analysis were subjected to multivariate Cox regression analysis as covariates, tumor PD-L2 expression was found to be an independent predictor for 5-year OS in advanced stage colon carcinoma patients (Table 5). Patients with low tumor PD-L2 levels (HR 1.59, 95% CI 1.206–2.106, *p* = 0.001) presented an increased risk for poor OS after adjustment for sex, age, pT stage, pN stage, pTNM stage, LVI, PNI, tumor location and immune signatures. Moreover, stage III and IV patients who received postoperative chemotherapy with low tumor PD-L2 levels presented an increased risk for poor OS after adjustment for age, pT stage, LVI, PNI and tumor location (Fig. 2D,F). These results show that the level of tumor PD-L2 could independently predict the prognosis of advanced stage CRC, especially patients who received postoperative chemotherapy (Table 5).

## Discussion

In the present study, we found that tumor PD-L2 expression was positively associated with the status of tumor PD-L1 in colon carcinoma. However, tumor PD-L2 was inversely associated with intratumoral TIL densities in colon carcinoma, such as CD8^+^ TIL and PD1^+^ TIL densities, suggesting a possible role of tumor PD-L2 in suppressing antitumor immune responses in colon carcinoma. Moreover, tumor PD-L2 expression was remarkably associated with 5-year OS in advanced stage colon carcinoma, suggesting that it can be considered an independent prognostic factor for advanced stage colon carcinoma, especially for patients who receive postoperative chemotherapy.

CRCs are often heterogeneous tumors because of the accumulation of somatic mutations and the alternations in host–tumor interactions within the TME. Lymphocyte alterations such as intraepithelial T cell infiltration within the TME, have been associated with favorable survival outcomes in CRC^13–15^. Therefore, a combination of molecular characteristics and immune cell infiltration parameters, defined as “consensus molecular subtypes” (CMSs), has been proposed to classify CRCs^28^. Consistent with this classification, our previous studies found that strong positive correlations between PD-L1 and intratumoral CD8^+^ infiltration, and the infiltration of CD8^+^ TILs and tumor PD-L1 status were positively associated with clinical outcome in colorectal cancer^12,29^. Interferon-γ (IFN-γ) secreted by CD8^+^ T lymphocytes is required for PD-L1 upregulation^24^, serving as a compensatory feedback mechanism for the adaptive immune response within the TME^30,31^. Similarly, PD-L2 can be induced by the adaptive immune resistance mechanism, depending on the milieu of inflammatory cytokines in the tumor microenvironment; for example, IFN-γ, IL-4 and colony-stimulating factor 2 (CSF-2) upregulate PD-L2^11^. Although we observed a positive association between PD-L2 and PD-L1 expression, we found that tumor PD-L2 expression was inversely associated with the intratumoral infiltration of CD8^+^ immune cells, suggesting a unique immunologic role of tumor PD-L2 within the TME. Several studies have reported that tumor PD-L2 is inversely correlated with TIL density and lymphocyteic reaction alterations, leading to inhibition of anti-cancer immunity^32–35^. Ohigashi et al. indicated that *PD-L2* mRNA expression was inversely correlated with CD8^+^ TILs in patients with esophageal cancer^32^. Masugi et al. recently indicated that tumor PD-L2 expression was negatively correlated with the Crohn-like lymphoid reaction in CRC, suggesting that tumor PD-L2 may inhibit T cell maturation, leading to downregulation of the Crohn-like lymphoid reaction against colorectal carcinoma^33^. Sridharan et al. also showed that high tumor PD-L2 led to decreased expression of immune gene signatures within the TME via the β-catenin/Wnt and PI3K pathways in adenoid cystic carcinoma (ACC)^35^. These results suggest that tumor PD-L2 expression may inhibit anti-cancer immunity against colon carcinoma, implying that tumor PD-L2 might be upregulated by other mechanisms, such as hypoxia. Pinato et al. revealed that PD-L2 expression was upregulated by hypoxia-induced factor 1 (HIF-1)^36^, which contributed to immune resistance within the TME. Therefore, it is possible that PD-L2 may be regulated by HIF-1 to potentially mediate immune resistance by downregulating lymphocytic reactions to avoid immune responses within the TME^17,36,37^. However, whether PD-L2 causes the immune suppression within the TME and its molecular mechanism require further investigation in the future.

The association between PD-L2 expression and survival outcome has been investigated in several malignancies, including CRC, melanoma, pancreatic cancer, and esophageal cancer^33,38–41^; however, discrepancies between these studies have been observed. Several studies reported that high PD-L2 expression was associated with poor patient survival, including studies in esophageal cancer^32^, pancreatic ductal adenocarcinoma^42^ and colorectal cancer^38,39^, and high tumor PD-L2 expression was associated with a favorable survival outcome in melanoma^40^ and colorectal cancer^41^. However, Masugi et al. reported that there was no significant correlation between tumor PD-L2 expression and survival in CRC patients^33^. This discrepancy may be due to the lack of FDA-approved IHC antibodies and differences in the criteria between these studies, which could have led to variations in PD-L2 expression and survival results in CRC. The IHC results in these studies were dramatically different. Previous studies have shown that PD-L1 is expressed on tumor and stromal cells^43^; however, the expression of PD-L2 is controversial. Masugi et al. reported that 78% of stromal cells expressed PD-L2, and 51.6% of tumor cells expressed PD-L2^33^, while Guo et al. indicated 17.4% of immune cells expressed PD-L2 and 19.3% of tumor cells expressed PD-L2 in CRC^44^. Therefore, standardization of IHC antibodies and evaluation criteria is urgently needed. In the current study, we found that 30.9% of tumor cells and fewer immune cells expressed PD-L2. Based on our IHC results, we found that increased PD-L2 expression positively correlated with better overall survival in advanced stage colon carcinoma, which is consistent with the results from the TCGA database^20,25^. The RNA-seq results together with clinical information showed that *PD-L2* mRNA was an independent prognostic factor for colorectal cancer in data from the TCGA database. These results suggest that tumor PD-L2 is an independent prognostic factor for OS in advanced stage colon carcinoma, especially in patients who receive postoperative chemotherapy. However, there are several limitations in our study. First, for the assessment of the immune signatures and tumor PD-L2 status by multivariate logistic regression analyses, we cannot exclude the possibility that the role of chemokines within the TME may regulate the recruitment of immune cells. We did not measure chemokines in this retrospective study, and potential chemokine confounders need to be considered in future studies. Second, based on our results, we speculate that tumor PD-L2 may inhibit intratumoral T cell infiltration. However, we did not elucidate what intrinsic and extrinsic factors contribute to PD-L2 upregulation. Further studies are necessary to address possible molecular mechanisms for tumor PD-L2 signaling, intrinsic factors, extrinsic factors and immune cell infiltration.

In addition, a recent study reported that not only the PD-L1 status but also the PD-L2 status was useful for predicting the clinical response to anti-PD1 immunotherapy in head and neck carcinoma^45^, suggesting that the status of tumor PD-L2 may have clinical implications for anti-PD1 immunotherapy. However, further studies are necessary to investigate the prognostic value of PD-L2 expression. Although both PD-L1 and PD-L2 proteins share the same receptor, PD-1, other receptors for PD-L2 (RGMb) have been reported^46^, suggesting that the role of PD-L2 in suppressing T cell responses may be different. Nonetheless, our results showed that the status of PD-L2 was not associated with MMR deficiency, strongly suggesting that further investigation to clarify the potential molecular mechanism of PD-L2 expression is necessary^47^. Accordingly, further investigations of PD-L2 stratifying patients according to receipt of anti-PD1/PD-L1 immunotherapy are needed.

Taken together, our results showed that tumor PD-L2 expression was inversely associated with the intratumoral infiltration of CD8^+^ TILs in advanced-stage colon carcinoma, suggesting a possible influence of PD-L2-expressing tumor cells on adaptive antitumor immunity. Moreover, patients with elevated tumor PD-L2 levels had favorable survival outcomes, suggesting that tumor PD-L2 may be an independent prognostic factor for advanced stage colon carcinoma. Since the inflammatory microenvironment in the gastrointestinal tract plays a role in CRC progression via host-immunity interactions^48^, the upregulation of tumor PD-L2 may be due to adaptive immunity, suggesting that PD-L2 may have prognostic value and can be considered a therapeutic target for immunotherapy in colon carcinoma.

## Abbreviations

CRC: Colorectal cancer
CMS: Consensus molecular subtype
LVI: Lymphovascular invasion
MSI: Microsatellite instability
OS: Overall survival
PD-L1: Programmed cell death 1 ligand 1
PD-L2: Programmed cell death 1 ligand 2
PNI: Perineural invasion
TILs: Tumor-infiltrating lymphocytes
TMA: Tissue microarray
TME: Tumor microenvironment
